# Krüppel-like factor 4 promotes survival and expansion in acute myeloid leukemia cells

**DOI:** 10.18632/oncotarget.27878

**Published:** 2021-02-16

**Authors:** Andrew Henry Lewis, Cory Seth Bridges, Viraaj Singh Punia, Abraham Fausto Jornada Cooper, Monica Puppi, H. Daniel Lacorazza

**Affiliations:** ^1^Department of Pathology & Immunology, Baylor College of Medicine, Texas Children’s Hospital, Houston, TX 77030, USA; ^2^SMART Program at Baylor College of Medicine Houston, Houston, TX 77030, USA

**Keywords:** acute myeloid leukemia, KLF4, cancer, gene editing, cell growth

## Abstract

Acute myeloid leukemia (AML) is an aggressive hematological malignancy of the bone marrow that affects mostly elderly adults. Alternative therapies are needed for AML patients because the overall prognosis with current standard of care, high dose chemotherapy and allogeneic transplantation, remains poor due to the emergence of refractory and relapsed disease. Here, we found expression of the transcription factor KLF4 in AML cell lines is not silenced through *KLF4* gene methylation nor via proteasomal degradation. The deletion of *KLF4* by CRISPR-CAS9 technology reduced cell growth and increased apoptosis in both NB4 and MonoMac-6 cell lines. Chemical induced differentiation of gene edited NB4 and MonoMac6 cells with ATRA and PMA respectively increased apoptosis and altered expression of differentiating markers CD11b and CD14. Transplantation of NB4 and MonoMac-6 cells lacking *KLF4* into NSG mice resulted in improved overall survival compared to the transplantation of parental cell lines. Finally, loss-of-KLF4 did not alter sensitivity of leukemic cells to the chemotherapeutic drugs daunorubicin and cytarabine. These results suggest that *KLF4* expression supports AML cell growth and survival, and the identification and disruption of KLF4-regulated pathways could represent an adjuvant therapeutic approach to increase response.

## INTRODUCTION

Acute myeloid leukemia (AML) is a malignant hematological disease that arises from genetic lesions in hematopoietic cells early in the hierarchy of normal myelopoiesis and a difficult to treat leukemia because of complexity of mutations and chromosomal translocations. Another complicating factor is the dynamic expansion of leukemic clones in the course of disease progression and in response to selective pressure imposed by antineoplastic drugs. Morphologic, cytogenetic, and genome sequence studies have been useful for the classification and treatment stratification of AML patients. The standard of care drugs, such as daunorubicin and cytosine β-D-arabinofuranoside (ara-C), non-selectively eliminate dividing cells, and can result in the outgrowth of aggressive leukemic clones that drive drug resistance and relapse [1, 2]. For the development of curative treatments, the identification of genes involved in AML pathogenesis will lay the groundwork for designing novel targeted therapies.

The Krüppel-like factor 4 (KLF4) gene is located on human chromosome 9q31.2 and encodes for a transcription factor with key stemness properties better recognized as one of the factors regulating reprogramming of adult human fibroblasts into induced pluripotent stem cells [3, 4]. KLF4 exerts its functions in cell division, survival, and differentiation through various gene regulation mechanisms, including interactions with the histone modifying enzymes p300 [5, 6] and histone deacetylases [7, 8], maintaining chromatin structures [9, 10], and binding to both methylated and non-methylated DNA [11, 12]. In hematopoietic cells, KLF4 has important functions in the regulation of hematopoiesis, such as regulation of monocyte development while restraining granulocyte production [13], survival of natural killer cells [14], and restriction of homeostatic and antigen driven T cell proliferation [15, 16]. The role of KLF4 in hematological malignancies has not been clearly defined because of its dual role as tumor suppressor and an oncogene depending on the leukemic cell context [17]. Supporting a suppressive role in leukemia, KLF4 inhibits T-cell acute lymphoblastic leukemia (T-ALL) and KLF4 expression is silenced in pediatric T-ALL by CpG promoter methylation, which was associated with aberrant expression of MAP2K7, a kinase pathway that accelerates disease progression [18, 19]. Similarly, low expression levels found in B-cell non-Hodgkin and Hodgkin lymphomas, multiple myeloma, CDX2-driven AML, and FOXC1 positive AML hematopoietic stem/progenitor cells suggest a leukemic suppressive function [20–24]. However, other findings highlight a potential pro-leukemic capacity of KLF4. For example, high KLF4 levels have been associated with poor prognosis in pediatric Burkitt lymphoma [25] and KLF4 was found essential for the survival of leukemic stem cells via repression of the DYRK2 gene that encodes a kinase involved in the regulation of p53 activity and c-Myc stability [26]. Particularly in AML, there are contrasting evidences in the role of KLF4. The lack of mutations and genetic alterations suggest that KLF4 may be required for disease progression [27] whereas studies using the Cancer Genome Atlas have shown that KLF4 expression is not uniform among AML patients, but is generally lower than normal blood cells [7, 28] and associated with leukemia stage [22, 28, 29]. Although these findings have fueled a presumption of tumor-suppressive function for KLF4 in AML, a model of ZMYM2-FGFR1 leukemia suggests KLF4 may be involved in AML leukemogenesis [30]. Altogether, these observations highlight a need to better define the role of KLF4 in leukemia and in particular AML.

Here, we report that KLF4 promotes survival and expansion of AML cells. KLF4 is expressed in a panel of AML cell lines, which correlates with absence of *KLF4* gene methylation and post-translational proteasomal degradation. Further, *KLF4* gene deletion via CRISPR-Cas9 technology in NB4 and MM6 cell lines resulted in impaired cell growth and survival and significant longer latency when transplanted into NSG mice. Additionally, targeting of KLF4 regulated pathways as adjuvant therapy is possible because *KLF4* deletion did not affect sensitivity to daunorubicin and ara-C. These findings suggest targeting of KLF4 or KLF4-regulated pathways as a novel alternative to control leukemia burden in AML and further mechanistic studies to identify associated potential targets are warranted.

## RESULTS

### Regulation of KLF4 expression in AML cell lines

**Figure 1:**
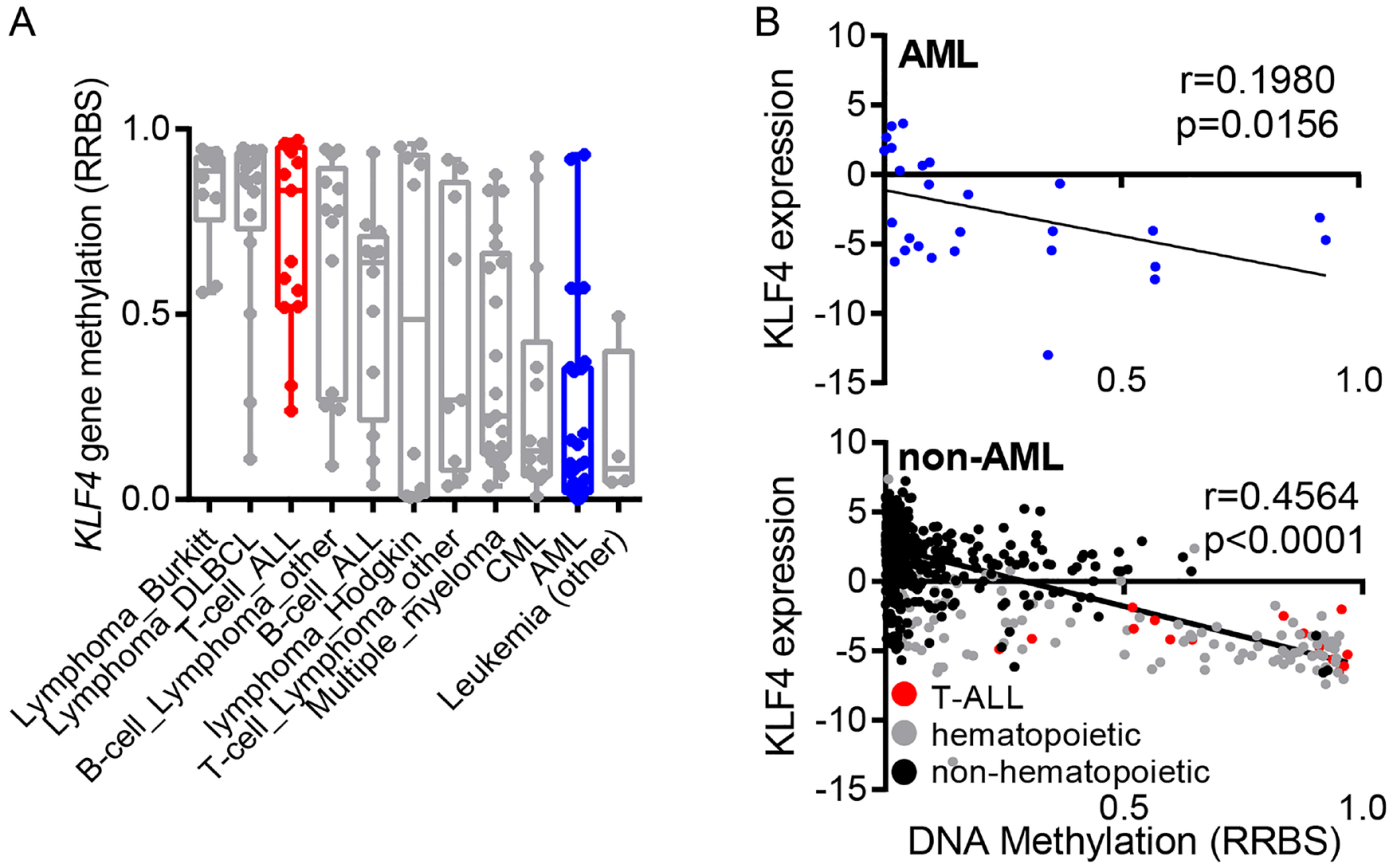
Regulation of KLF4 expression in leukemic cell lines. (**A**) Analysis of KLF4 gene methylation from the Cancer Cell Line Encyclopedia (CCLE) in a panel of hematologic malignancy cell lines. (**B**) Correlation of KLF4 transcript levels and DNA methylation in AML and non-AML cell lines. Linear regression analysis was conducted with 95% confidence interval.

In order to compare CpG DNA methylation in the human *KLF4* gene across diverse hematologic malignancies, we analyzed publicly available reduced representation bisulfite sequencing (RRBS) data through the cancer cell line encyclopedia (CCLE). This analysis demonstrated that the *KLF4* gene is hypomethylated in most AML cell lines (Figure 1A). In contrast, T-cell acute lymphoblastic leukemia (T-ALL) showed elevated gene methylation as previously described by our group [19]. Further analysis of KLF4 transcript levels and DNA methylation revealed no significant correlation in AML while the levels of KLF4 correlated with gene methylation in other types of cancer (Figure 1B). This analysis suggests that KLF4 expression is not silenced epigenetically by DNA methylation at least in AML cell lines.

**Figure 2:**
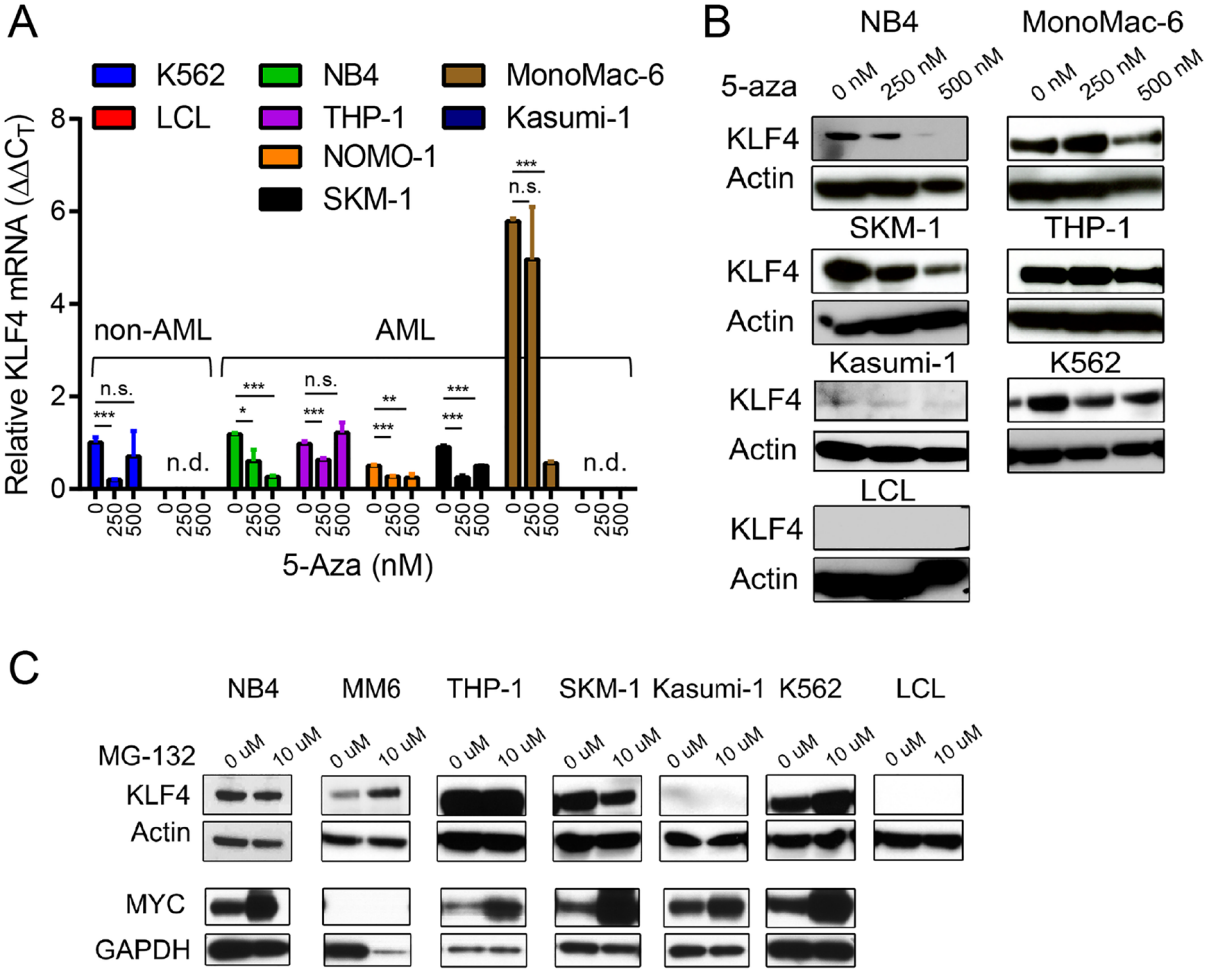
Epigenetic and post-translational regulation of KLF4 in AML cell lines. (**A**) Relative KLF4 expression was measured by qPCR in AML, CML, and LCL cell lines cultured in the presence of 250 and 500 nM 5-Aza for 96 hours to induce DNA demethylation. Data represents relative mRNA expression (DDC_T_) expressed as mean ± s.d. (*n* = 3). (**B**) Immunoblot analysis of KLF4 expression after treatment with 5-Aza for 96 hours. Actin was used as a loading control. (**C**) Immunoblot analysis of KLF4 and MYC expression in cell lines treated with 10 μM of MG-132 for 4 hours. Actin and GAPDH were used as loading control. Data shown are representative of two independent experiments. ^*^ *p* < 0.05, ^**^ *p* < 0.01, ^***^ *p* < 0.001, two-tailed Student’s *t*-test.

To further evaluate whether DNA methylation is involved in the regulation of KLF4 expression, we treated a panel of AML (NB4, THP-1, MonoMac-6, SKM-1), CML (K562), and EBV-transformed lymphoblastoid (LCL) cell lines with the hypomethylating agent 5-Azacytidine (5-aza). Neither KLF4 transcripts measured by qPCR nor protein levels detected by immunoblots demonstrated induction of KLF4 levels upon treatment with 250 and 500 nM 5-aza (Figure 2A and 2B). To examine post-translational regulation of KLF4, treatment of AML cell lines with the proteasome inhibitor MG-132 (10 μM) revealed KLF4 is not actively proteolyzed by the proteasome (Figure 2C). Collectively, this data is consistent with the analysis of human AML CCLE data and supports our assessment that KLF4 is not being actively repressed by AML cells. To evaluate the importance of KLF4 expression in AML, we selected the NB4 and MonoMac-6 (MM6) cell lines for further study, which represent the range of KLF4 expression found in our panel as well as distinct AML subtypes.

### *KLF4* gene deletion impairs cell growth and promotes differentiation and apoptosis

**Figure 3:**
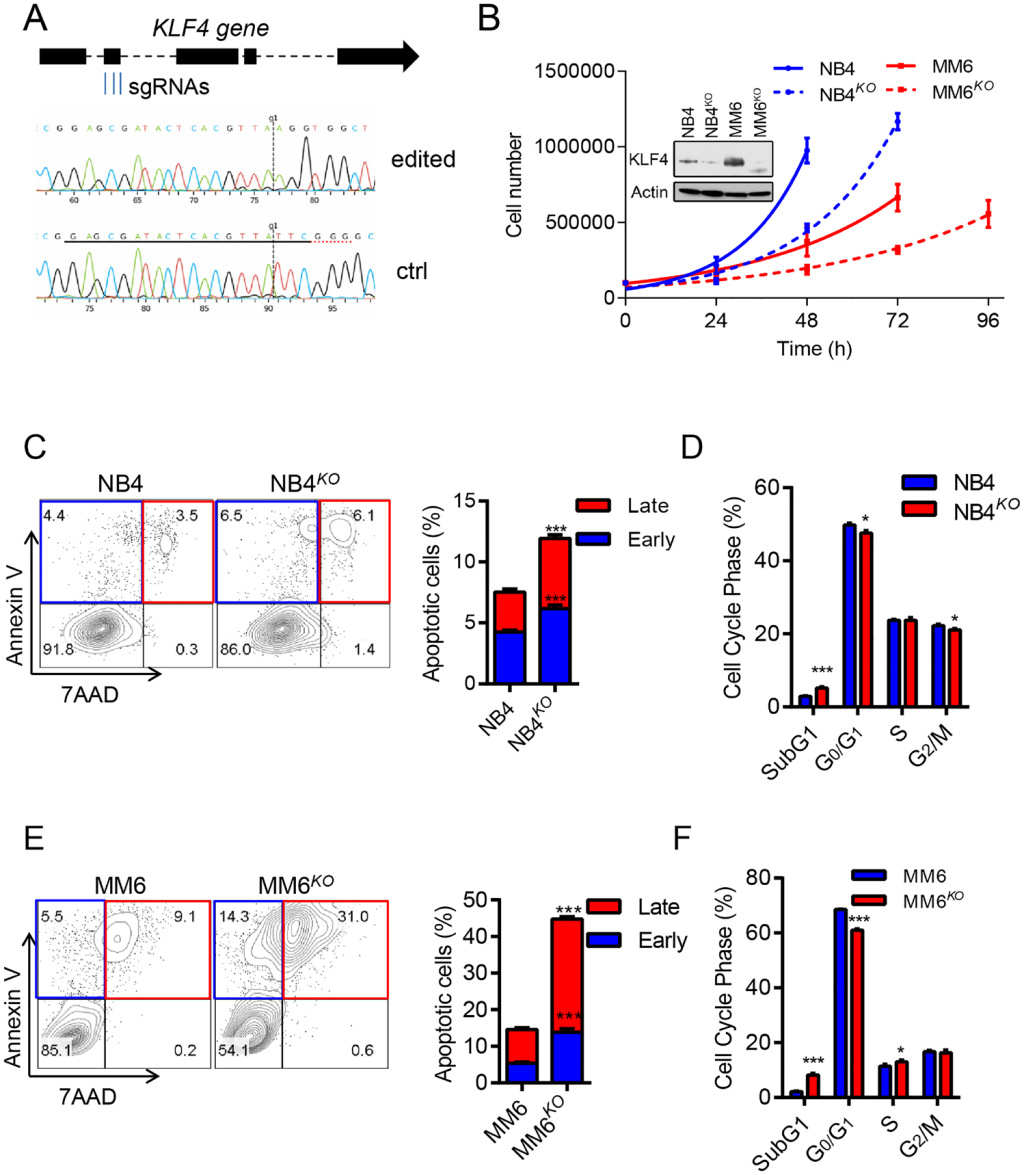
KLF4 deletion inhibits cell growth and induces apoptosis. (**A**) Multiplex sgRNA targeting strategy to induce KLF4 gene deletion via CRISPR/CAS9 technology and comparison of sequencing tracks of parental and edited leukemic cells. (**B**) Cell growth of NB4^*KO*^ and MM6^*KO*^ cells compared to parental cell lines (*n* = 3); inset shows loss of KLF4 expression in edited cell lines. (**C**) Flow cytometric detection of Annexin V and 7AAD in NB4 and NB4^*KO*^ cells. Representative plots are included on the left and statistical analysis on the right (*n* = 3). (**D**) Cell cycle analysis of NB4 and NB4^*KO*^ by flow cytometric detection of propidium iodine staining of nuclei (*n* = 3). (**E** and **F**) Apoptosis and cell cycle analysis for MM6 and MM6^*KO*^ cells. Data represent mean ± s.d. (*n* = 3). ^*^ *p* < 0.05, ^***^ *p* < 0.001, two-tailed Student’s *t*-test.

To address the significance of KLF4 expression in AML, we deleted the *KLF4* gene in the MM6 cell line, which represents *MLL*-rearranged leukemia, and the NB4 cell line which represent *PML-RARα* PML using CRISPR-Cas9 technology. MM6 cells expressed relatively higher levels of KLF4, transcript and protein, than NB4 cells (Figure 3B, Supplementary Figure 1A and 1B). Multiplex sgRNA designed to target the second exon of the *KLF4* gene were nucleofected into cells and single-cell clones were generated via cell dilution (Figure 3A). KLF4 knockout clones (NB4^*KO*^ and MM6^*KO*^) were confirmed by DNA sequencing and immunoblots (Supplementary Figure 2A and 2B). Analysis of cell growth demonstrated that *KLF4* loss diminished expansion in both MM6 and NB4 cell lines (Figure 3B). This was caused by increase in apoptosis in NB4 cells and MM6 cells (up 4-fold) (Figure 3C and 3E). Cell cycle analysis revealed increased sub-G1, consistent with increased apoptosis, and small reduction in G0/G1 phase of the cell cycle (Figure 3D and 3F). Of note, multiple clones for each cell line were analyzed. These data suggest that KLF4 supports growth and survival in two AML cell lines encompassing different driver translocations.

**Figure 4:**
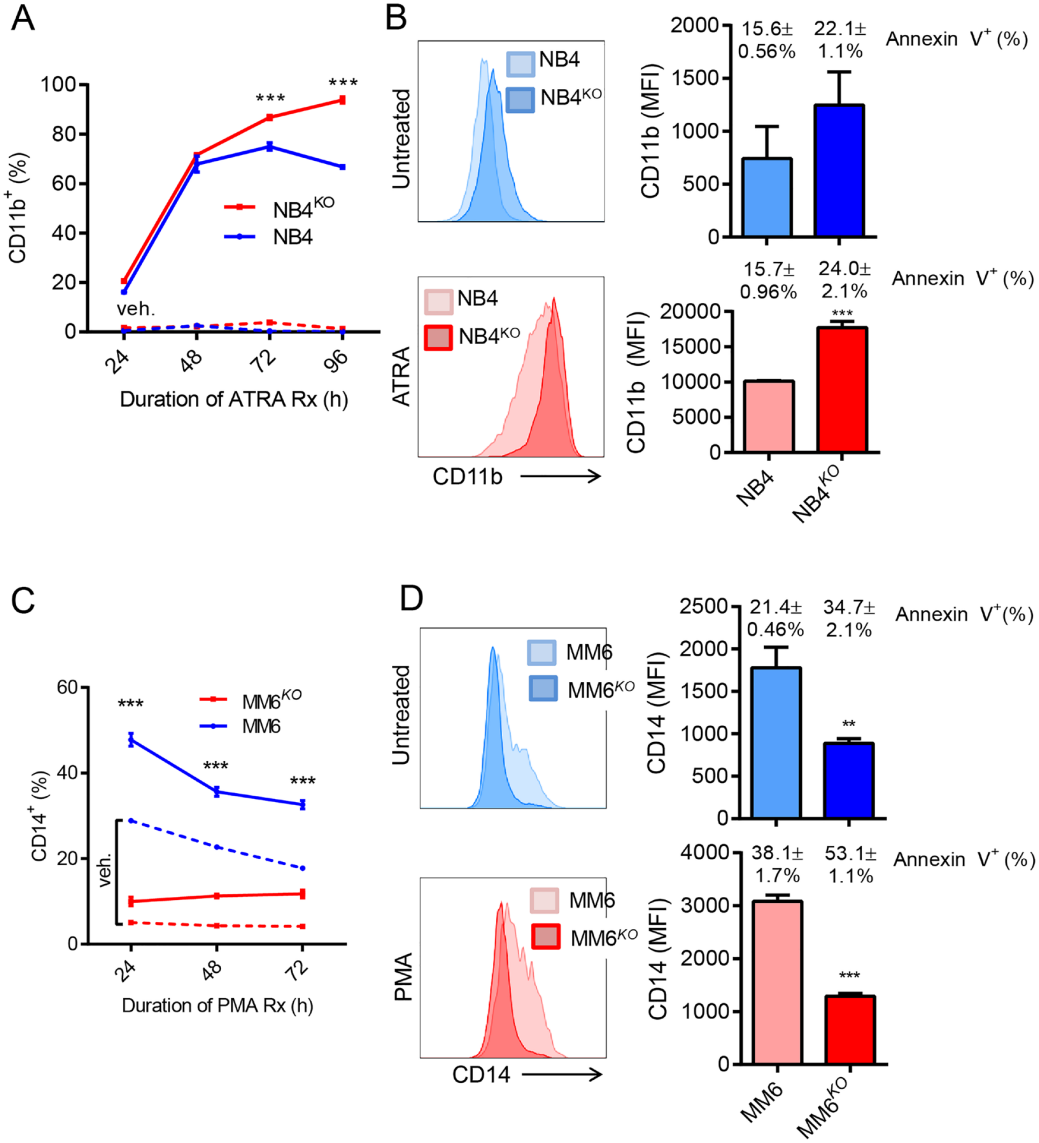
Role of KLF4 in chemical induced differentiation of AML cell lines. (**A**) Frequency of CD11b expression was monitored in NB4 and NB4^*KO*^ cells by flow cytometry at different culture times with 3 μM ATRA (*n* = 3). Dashed line indicates vehicle control. (**B**) Expression of CD11b in NB4 and NB4^*KO*^ cell lines with and without ATRA treatment. Bar graph shows MFI and the percentage of apoptosis measured as annexin V positive cells. Data represent mean ± s.d. (*n* = 3) (**C**) Frequency of CD14 expression was monitored in MM6 and MM6^*KO*^ cells by flow cytometry at different culture times with 3 nM PMA (*n* = 3). Dashed line indicates vehicle control. (**D**) Expression of CD14 in MM6 and MM6^*KO*^ cells with and without PMA treatment. Bar graph shows MFI and the percentage of apoptosis measured as annexin V positive cells. Data represent mean ± s.d. (*n* = 3). Data represent mean ± s.d. ^**^ *p* < 0.01, ^***^ *p* < 0.001, two-tailed Student’s *t*-test.

Because KLF4 is known to play a role in myeloid differentiation [13, 28, 31–34], we next sought to evaluate whether KLF4 regulates both differentiation and apoptosis. We incubated NB4 and NB4^*KO*^ cells with all-trans retinoic acid (ATRA) at a concentration of 3 μM and evaluated differentiation via flow cytometric detection of cell surface expression of CD11b. NB4^*KO*^ cells showed increased expression of CD11b after 48 hours incubation with ATRA with a moderate increase in apoptosis (Figure 4A and 4B). As MM6 represents a monocytic leukemia subtype, we treated MM6 and MM6^*KO*^ cells with 3 nM phorbol 12-myristate 13-acetate (PMA) and evaluated expression of CD14. MM6^*KO*^ displayed lower CD14 expression than vehicle control (15–25% in control) and the difference became more significant after PMA treatment, which also correlated with increased apoptosis (Figure 4C and 4D). Taken together, these results support a model in which apoptosis induced by lack of KLF4 correlates with cell differentiation depending on the cell line under study.

### KLF4 expression shortens latency of *in vivo* AML xenografts

**Figure 5:**
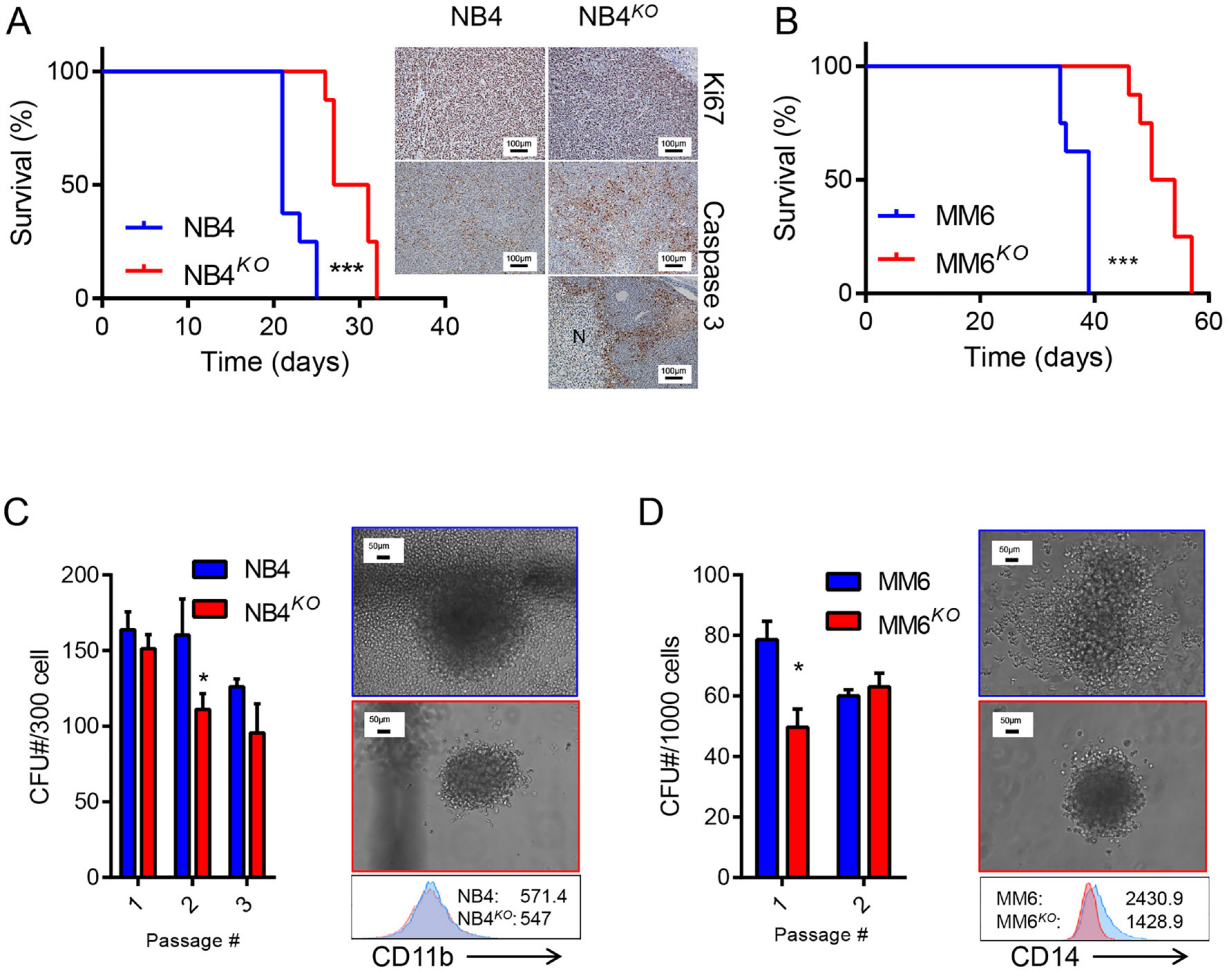
Loss of KLF4 reduces leukemia burden in a cell-based xenograft model. (**A**) Overall survival of NSG mice transplanted with 5 × 10^6^ NB4 or NB4^*KO*^ cells (*n* = 8/group) is shown as Kaplan Maier curves. Tumors from moribund mice were analyzed by immunohistochemistry of Ki67 and Caspase 3. Bar represents 100 μm and N a necrotic area. (**B**) Overall survival of NSG mice transplanted with 5 × 10^6^ MM6 or MM6^*KO*^ cells (*n* = 8/group) is shown as Kaplan Maier curves. (**C**) Colony forming capacity of NB4 and NB4^*KO*^ cells in methylcellulose medium. (**D**) Colony forming capacity of MM6 and MM6^*KO*^ cells in methylcellulose medium. In C and D, representative images of colonies and immunophenotype (MFI) of cells collected from methylcellulose during passage are shown (blue: parental cell line; red: KO). Data in C and D represent mean ± s.d. ^*^ *p* < 0.05, two-tailed Student’s *t*-test (C and D) and ^***^ *p* < 0.001, Log-rank test (A and B).

After observing the effects of *KLF4* deletion on cell growth and survival in AML cell lines, we sought to evaluate the importance of KLF4 in *in vivo* leukemic expansion. We monitored overall survival of NSG mice transplanted with NB4, NB4^*KO*^, MM6, and MM6^*KO*^ cells. Consistent with *in vitro* data, mice receiving NB4^*KO*^ and MM6^*KO*^ cells had significantly prolonged survival compared to corresponding parental cell line (Figure 5A and 5B). As reported previously, NB4 cells form tumors in the ovary when transplanted into female NSG mice [35, 36] and histologic analysis of NB4^*KO*^ tumors revealed large necrotic regions surrounded by apoptotic cells (Figure 5A). Quantification of immunohistochemistry showed no significant differences in Ki67 positive cells whereas the percentage of caspase 3 positive cells increased in NB4^*KO*^ compared to NB4 (not shown). NB4^*KO*^ and MM6^*KO*^ cells cultured in methylcellulose medium showed smaller and more compact colonies with reduced colony-forming capacity (Figure 5C and 5D). While NB4 cells did not upregulate CD11b expression in methylcellulose culture, MM6 cells demonstrated induction of CD14 expression. This induction was impaired in MM6^*KO*^ cells (Figure 5C and 5D), consistent with response to treatment with PMA (Figure 4C and 4D). Collectively, these data suggest pharmacological inhibition of KLF4 downstream targets may represent an alternative to control leukemia burden.

### KLF4 does not regulate sensitivity to drugs used in AML therapy

**Figure 6:**
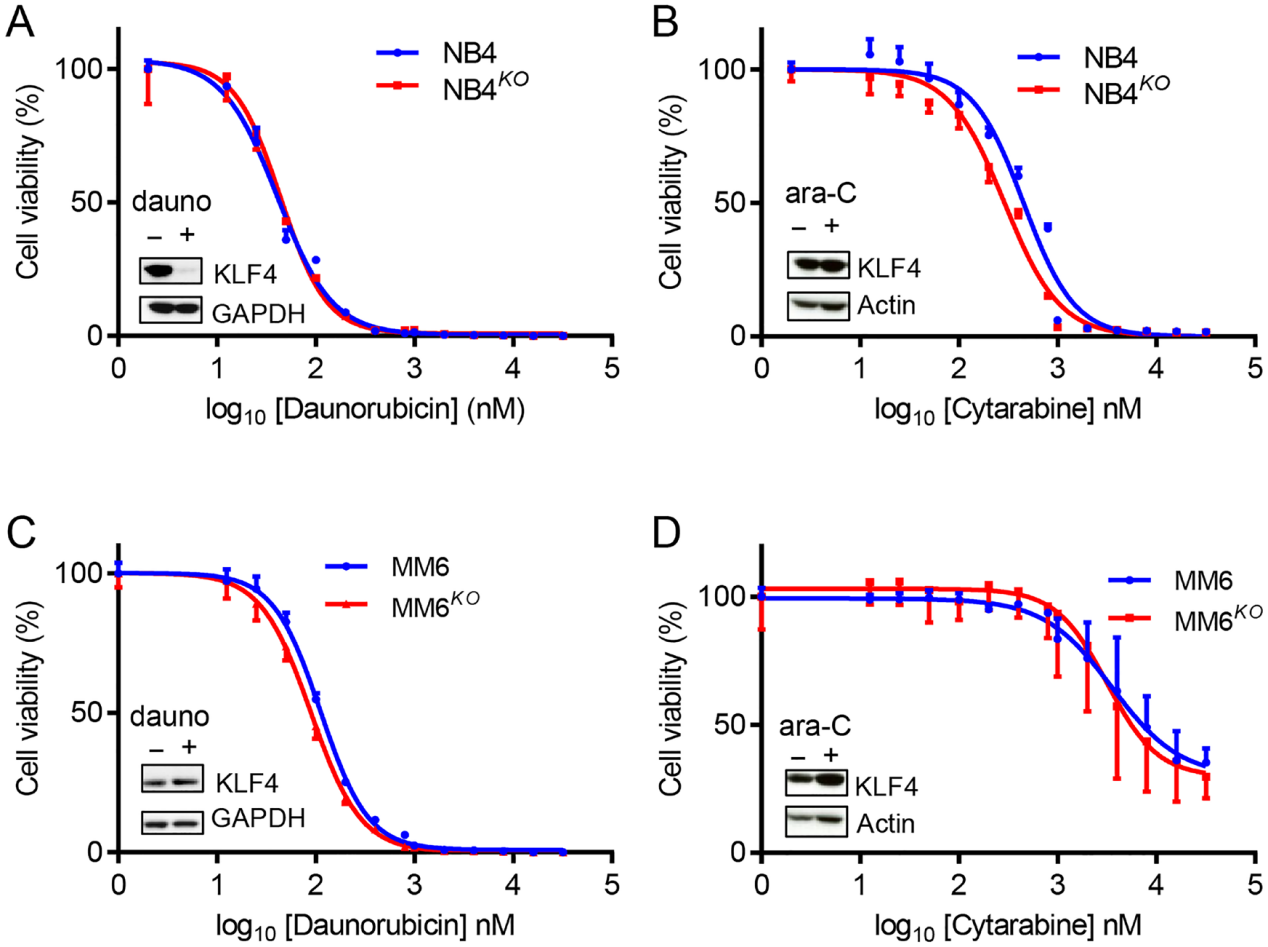
KLF4 deletion does not alter sensitivity to standard chemotherapeutic drugs. Cytotoxicity was determined using Cell-Titer Glo cytotoxicity assay after 48 h treatment with either daunorubicin or ara-C. (**A**) Cytotoxicity of NB4 and NB4^*KO*^ cells to daunorubicin (*n* = 3). (**B**) Cytotoxicity of NB4 and NB4^*KO*^ cells to ara-C (*n* = 3). (**C**) Cytotoxicity of MM6 and MM6^*KO*^ cells to daunorubicin (*n* = 3). (**D**) Cytotoxicity of MM6 and MM6^*KO*^ cells to ara-C (*n* = 3). Expression of KLF4 protein in parental cells in response to treatment at IC_50_ dose is shown in figure insets. Data represent mean ± s.d.

To evaluate whether targeting of KLF4 might interfere with standard-of-care chemotherapies, we studied the role of KLF4 expression in response to daunorubicin and ara-C drugs used in AML therapy. Of note, NB4 cells treated with daunorubicin displayed loss of KLF4 protein, while all other cell line-drug combinations were unchanged (Figure 6A–6D). When we treated parental and KLF4 knockout cell lines with increasing doses of daunorubicin or ara-C, we found the absence of KLF4 did not significantly alter response to these compounds, with the exception of NB4 cells treated with ara-C (Figure 6A–6D). To evaluate pharmacological upregulation of KLF4, we tested APTO-253, a compound previously reported to induce cytotoxicity through KLF4 [37]. However, deletion of KLF4 in NB4 cells did not alter APTO-253-induced cytotoxicity (Supplementary Figure 3A–3D), suggesting a KLF4 independent mechanism, likely by loss of MYC [38]. Finally, we treated gene edited cell lines with the BCL2 inhibitor ABT-199 (Venetoclax) to test whether *KLF4* deletion might impact induction of apoptosis. NB4^*KO*^ and MM6^*KO*^ cells showed a dose-dependent cytotoxicity comparable to controls (Supplementary Figure 4A and 4B). Taken together, inhibition of KLF4 should not significantly affect the course of treatment with standard-of-care chemotherapy and warrants further studies to elucidate ‘druggable’ targets regulated by KLF4.

## DISCUSSION

Currently, the response of AML patients to standard multi-drug therapy has not significantly improved through the years because of complex genetic heterogeneity and therapy-induced generation of more aggressive leukemic clones that are resistant to drug therapy. The mutational landscape determined by genome sequencing revealed the presence of multiple driver mutations in each patient [39, 40]; however, these studies cannot identify genes with pro-leukemic functions. This work identifies that the transcription factor KLF4 contributes to expansion of AML cells and in turn loss-of-KLF4 reduces leukemic cell growth and survival.

Proteins with tumor suppressor function are inactivated in cancer through DNA methylation, gene mutation/deletion, or post-translational proteolysis [41–43]. COSMIC data analysis failed to identify mutations in the *KLF4* gene [27]. Although a 9q31 breakpoint has been described in a pediatric MDS patient [44], the functional contribution of KLF4, if any, present in this case is unknown. Our findings support a different function for KLF4 by promoting the expansion of AML cells. More consistent with a pro-oncogenic role of KLF4 in AML, DNA methylation did not appear to play a significant role in AML cell lines, in contrast to other cancer types [45, 46]. Similarly, KLF4 is not proteolyzed by the proteasome and therefore is expressed in a panel of AML cell lines. Some efforts have been made to explore the effect of KLF4 expression in AML; however, these studies have been carried out via ectopic KLF4 expression that aberrantly causes differentiation and cell death, including via interactions with BAALC and HDACs, and induction of microRNAs [7, 28, 47]. This approach, while in some cases mechanistically informative, leaves a gap in understanding for the function of KLF4 expression in leukemic cells.

KLF4 shows higher expression in monocytic leukemia compared to other AML subtypes, which comprise M4 and M5 AMLs in the FAB classification system and frequently feature *MLL-*rearrangements [48]. Although KLF4 is known to regulate monocytic development, the function of KLF4 expression in monocytic leukemia has not been thoroughly explored [13]. The MM6 cell line represents a monocytic AML containing the *MLL-AF9*-rearrangement and high levels of KLF4 expression relative to other tested cell lines. Interestingly, deletion of the KLF4 gene diminished cell growth and survival not only in this MLL-rearranged cell line, but also in NB4, a *PML-RARα*-rearranged cell line corresponding to the M3 subtype of AML, which express lower KLF4 levels compared to other AML cell lines [49, 50]. Gene edited NB4 and MM6 showed reduced clonogenicity and smaller colonies in methylcellulose cultures correlating with the reduction of proliferation and survival seen *in vitro* and in the xenograft model, suggesting that KLF4 likely plays a general role in AML regardless of the subtype. While it is promising that *KLF4* deletion is disruptive in AML cells with different cytogenetics, a more comprehensive study of downstream targets in a larger cohort of patients would be necessary to evaluate whether inhibiting KLF4 activity could represent a treatment modality applicable to most AML patients. It will be important to define also whether KLF4 is upregulated in leukemic clones emerging in refractory or relapsed leukemia because high levels of expression of KLF4 were found in leukemic clones in a relapsed patient with chronic lymphocytic leukemia [51].

Potential application of pharmacological inhibition of KLF4 regulated pathways in AML as adjuvant therapy to increase treatment response in patients will require further studies using patient samples. We tested whether genetic loss of KLF4 altered response to daunorubicin and cytarabine, two conventional chemotherapeutic agents used in standard of care of AML patients [52]. These chemotherapies are not well tolerated in the elderly patient population comprising AML, with median survival for patients on chemotherapy about 13 months for those 65–75 years of age, and only 6 months for 75 and older [53]. Data show that NB4 and MM6 cells responded to both drugs similarly in the presence or absence of KLF4. This is important because potential use of KLF4 inhibition as adjuvant therapy would require no interference with current therapies. Although no additive or synergistic effect of genetic loss of KLF4 was observed, pharmacological inhibition of downstream targets will be more adequate for pharmacological inhibition.

Collectively, genetic loss of KLF4 in AML cell lines provides novel insights into the importance of KLF4 in the maintenance of AML, particularly supporting leukemic cell growth and survival. Further studies are warranted to identify KLF4-regulated pathways amenable for therapeutic targeting with small molecules.

## MATERIALS AND METHODS

### Analysis of data from cancer cell line encyclopedia

KLF4 mRNA expression (RNASeq) and DNA methylation (RRBS) were downloaded for cell lines from the publicly accessible cancer cell line encyclopedia database from Broad Institute. Data were replotted and formatted using GraphPad prism software, and linear regression with coefficient of correlation was calculated. Data was filtered for cell lines comprising hematologic malignancy for visual representation.

### Cell lines and cell culture

SKM-1 was obtained from the DSMZ German collection of microorganisms and cell cultures. K562, U937, Kasumi-1 were obtained from the American Type Culture Collection (ATCC). NB4 cells were provided by Dr. Michele Redell (Baylor College of Medicine). THP-1 and MonoMac-6 cells (MM6) were provided by Dr. James Versalovic (Baylor College of Medicine). LCL cells were generated through transformation of B-cells using Epstein Barr Virus. NB4, K562, U937 and LCL were maintained in RPMI 1640 medium (Lonza) containing 10% fetal bovine serum (FBS), 2 mM L-glutamine. KASUMI-1 and SKM-1 cells were maintained in RPMI 1640 medium (Lonza) containing 20% fetal bovine serum (FBS), 2 mM L-glutamine. THP-1 cells were maintained in RPMI 1640 medium (Lonza) containing 10% fetal bovine serum (FBS), 0.05 mM β-mercaptoethanol according to ATCC recommendations. MonoMac-6 cells were maintained in RPMI 1640 medium (Lonza) containing 10% fetal bovine serum (FBS), 0.1 mM NEAA (Lonza), and OPI media supplement Hybrimax (1 mM oxaloacetate, 0.45 mM pyruvate, 0.2 U/ml insulin) (Sigma). The cell lines were authenticated (Cell Line Characterization, MD. Anderson) every 6 months and periodically tested for mycoplasma.

### Chemical compound treatment

For hypomethylating treatment, 5-aza (Sigma) was prepared in DMSO to a concentration of 100 mM and then diluted to final concentration by diluting in respective medium for each cell line. Cells were plated in 6-well plates (1.25 × 10^5^ cells/ml) and 5-aza was replaced every 24 hours for a total of 96 hours treatment. For proteasomal inhibition, 20 mM InSolution™ MG-132 (CalBioChem) was stored at −20°C. Cells were plated in 6-well plates (5.00 × 10^5^/ml) in the presence of 10 μM MG-132 for 4 h.

### Quantitative real-time PCR

Total RNA was extracted using the RNeasy Mini kit (Qiagen) from human AML cell lines. cDNA was synthesized from 100–500 ng RNA using oligoDT primers and a SuperScript III kit (Invitrogen). LightCycler-FastStart DNA Master SYBR Green I was used for quantitative real-time PCR as specified by the manufacturer (Roche Molecular Biochemicals). Reactions were performed using the StepOnePlus Real-Time PCR System (Applied Biosystems). Relative expression was normalized to β-actin expression (ΔΔC_T_). The following primers were used:

**Table:** 

Human KLF4	Forward	ATCTTTCTCCACGTTCGCGTCTG
	Reverse	AAGCACTGGGGGAAGTCGCTTC
Human β-actin	Forward	GCTCGTCGTCGACAACGGCTC
	Reverse	CAAACATGATCTGGGTCATCTTCTC

### Immunoblot analysis

Cell pellets were lysed with SDS Lysis Buffer (1% SDS, 10 mM Tris pH 7.4, 1 mM phenylmethylsulfonyl fluoride) and supplemented with Halt Protease and Phosphatase Inhibitor Cocktail (Thermo Fisher). Protein samples were resolved by SDS-PAGE (Novex NuPage Bis Tris Gel; Invitrogen MiniGel Tank) and then transferred onto PVDF membranes (iBlot 2 system, Invitrogen). The following antibodies were used: KLF4 D1F2 from Cell Signaling, anti-Actin A5316 from Sigma, Direct-Blot™ HRP anti-β-actin Antibody, GAPDH Loading Control Monoclonal Antibody (GA1R), PARP antibody from Cell Signaling, and MYC antibody from Cell Signaling. Primary antibodies were used at 1:1,000 dilution. Primary actin antibody was used at 1:100,000 dilution, or HRP anti-β-actin at 1:300,000. HRP cross-linked secondary antibodies (anti-rabbit IgG, HRP-linked Antibody #7074 and anti-mouse IgG, HRP-linked Antibody #7076 from Cell Signaling) were detected by West Femto Maximum Sensitivity Substrate (Thermo Fisher) and Amersham Hyperfilm ECL (GE). All secondary antibodies are used at 1:15,000-1:30,000 dilution. Protein quantity was normalized based on housekeeping control bands.

### KLF4 gene editing via CRISPR/CAS9

To knockout the KLF4 gene in MM6 and NB4 cells, we used 3 chemically modified synthetic sgRNAs from Synthego with sequences GCCATGTCAGACTCGCCAGG, CGCCGGGCCAGACGCGAACG, and GAGCGATACTCACGTTATTC and followed the instructions obtained from the manufacturer to form the RNP complex with Cas9 (Cas9 plus sgRNAs). Briefly, the Cas9-sgRNA RNP in a total volume of 12 μl were electroporated using the Neon transfection system (Thermo Fisher Scientific) under the following conditions: 1400V, 10 ms, 3 pulse. Electroporated cells were cultured in growth medium for 3 days and then single cell clones were obtained by diluting cells to 0.5 cells/100 μl and plating in 96-well U-bottom plate for 2 weeks. Genomic DNAs were isolated and then used for PCR amplification using forward primer GTGTTATGTCCTGTCTGCCCAATT and reverse primer GTTTTGGCTTCGTTTCTTCTCTTC, spanning the Cas9-sgRNA cleavage site. PCR amplicons were then used for sequencing analysis using sequencing primer (CTTACCCTCGTTCAGTGGCTCTT) to identify the knockout efficiency using Inference of CRISPR Edits (ICE) which is a free and open source software tool that offers fast and reliable analysis of CRISPR editing data from Synthego. Knockout was additionally verified by immunoblotting and PCR detection of cleaved genomic DNA on 2.0% agarose gel.

### Cell death and cell cycle assays

For analysis of cell growth, NB4, NB4^*KO*^, MM6, and MM6^*KO*^ cells were plated at 100,000 cells/ml and collected after 24 hours. For apoptosis detection, these cells were stained with Annexin V and 7-AAD according to BD Apoptosis detection kit protocol (BD Biosciences). For cell cycle analysis, cells were lysed in hypotonic DNA-staining solution containing 0.1% sodium citrate, 0.1% Triton-X, 100 μg/ml RNAse A, 50 μg/ml propidium iodide in de-ionized water. All samples were plated in triplicate. Cells were analyzed in a BD FACSCanto instrument and FlowJo software.

### Chemical induced differentiation

10 mM PMA (Sigma) and 100 mM ATRA (Sigma) were dissolved in DMSO and stored at −20°C. Compounds were diluted to 3 nM (PMA) and 3 μM (ATRA) in respective media and cells were suspended at 100,000 cells/ml in 6-well non-tissue culture plates. Cell differentiation was detected by flow cytometry, staining with PE anti-human CD14 (BioLegend) and APC anti-mouse/human CD11b (BioLegend) from cells sampled from culture every 24 hours. All sample data was collected on BD FACSCanto instrument and analyzed using FlowJo software.

### Sensitivity to chemotherapeutic drugs

10 mM stocks of daunorubicin hydrochloride and cytosine β-D-arabinofuranoside (Sigma) were dissolved in de-ionized water and stored at −20°C. Compounds were diluted to the specified final concentration in their respective media. The volume of vehicle was always lower than 0.1% of the total volume, and the same dilution of vehicle in culture medium was used as the vehicle control. Cells were plated in 96-well plates (1.5 × 10^4^ cells in 100 μl per well) with increasing doses of drug (0–32 μM) for 48 hours. Cell viability was measured using CellTiter-Glo Luminescent Cell Viability Assay (Promega). The half-maximal inhibitory concentration (IC_50_) was calculated by nonlinear regression analysis using GraphPad software.

### AML cell line xenografts

NB4 and MM6 cells were washed with PBS and resuspended in medical grade physiologic saline solution. Cells (5 million) were injected into 10–20 week old NOD scid gamma (NSG) mice via tail vein. Mice were monitored in peripheral blood by cytometric detection of human CD45 to determine leukemic cell engraftment and disease progression, and mice were euthanized upon signs of moribundity.

### Human colony-forming cells (CFC) assay

From culture, NB4 and NB4^*KLF4KO*^ cells (300 cells) and MonoMac-6 and MonoMac-6^*KLF4KO*^ cells (1000 cells) were serially plated in methylcellulose medium (MethoCult H4434, Stemcell Technologies) every 7 days. After each passage, colonies were counted, imaged, and replated in fresh methylcellulose medium. MonoMac-6 colonies were counted, imaged, and re-plated on day 14. Cells were also isolated after plating and analyzed by flow cytometry for expression of CD11b and CD14.

### Statistical analysis

Experiments were performed without blinding and with no exclusion of samples. Linear regression was performed with GraphPad Prism software. An unpaired 2-tailed Student *t* test was used for statistical analysis. The survival of leukemic mice was visualized using Kaplan-Meier curves, and statistical significance was calculated using the log-rank test (GraphPad Prism). *P* values were determined using GraphPad software. Results with a *P value* < .05 were considered statistically significant.

## SUPPLEMENTARY MATERIALS



## Abbreviations

AML: acute myeloid leukemia
MM6: MonoMac-6
ara-C: β-D-arabinofuranoside
KLF4: Krüppel-like Factor 4
CRISPR: clustered regularly interspaced palindromic repeats
CML: chronic myeloid leukemia
T-ALL: T-cell acute lymphoblastic leukemia
KO: knockout
NSG: NOD-scid-gamma
ATRA: All-trans retinoic acid
HDAC: histone deacetylase
5-aza: 5-azacytidine
FAB: French American British
PMA: phorbol 12-myristate 13-acetate
APL: acute promyelocytic leukemia

## References

[1] Shlush LI , Zandi S , Mitchell A , Chen WC , Brandwein JM , Gupta V , Kennedy JA , Schimmer AD , Schuh AC , Yee KW , McLeod JL , Doedens M , Medeiros JJ , et al. Identification of pre-leukaemic haematopoietic stem cells in acute leukaemia. Nature. 2014; 506:328–33. 10.1038/nature13038. 24522528 PMC4991939

[2] Welch JS , Ley TJ , Link DC , Miller CA , Larson DE , Koboldt DC , Wartman LD , Lamprecht TL , Liu F , Xia J , Kandoth C , Fulton RS , McLellan MD , et al. The origin and evolution of mutations in acute myeloid leukemia. Cell. 2012; 150:264–78. 10.1016/j.cell.2012.06.023. 22817890 PMC3407563

[3] Takahashi K , Tanabe K , Ohnuki M , Narita M , Ichisaka T , Tomoda K , Yamanaka S . Induction of pluripotent stem cells from adult human fibroblasts by defined factors. Cell. 2007; 131:861–72. 10.1016/j.cell.2007.11.019. 18035408

[4] Takahashi K , Yamanaka S . Induction of pluripotent stem cells from mouse embryonic and adult fibroblast cultures by defined factors. Cell. 2006; 126:663–76. 10.1016/j.cell.2006.07.024. 16904174

[5] Evans PM , Zhang W , Chen X , Yang J , Bhakat KK , Liu C . Kruppel-like factor 4 is acetylated by p300 and regulates gene transcription via modulation of histone acetylation. J Biol Chem. 2007; 282:33994–4002. 10.1074/jbc.M701847200. 17908689

[6] Zhang R , Han M , Zheng B , Li YJ , Shu YN , Wen JK . Kruppel-like factor 4 interacts with p300 to activate mitofusin 2 gene expression induced by all-trans retinoic acid in VSMCs. Acta Pharmacol Sin. 2010; 31:1293–302. 10.1038/aps.2010.96. 20711222 PMC4012908

[7] Huang Y , Chen J , Lu C , Han J , Wang G , Song C , Zhu S , Wang C , Li G , Kang J , Wang J . HDAC1 and Klf4 interplay critically regulates human myeloid leukemia cell proliferation. Cell Death Dis. 2014; 5:e1491. 10.1038/cddis.2014.433. 25341045 PMC4237257

[8] Hu C , Liu M , Zhang W , Xu Q , Ma K , Chen L , Wang Z , He S , Zhu H , Xu N . Upregulation of KLF4 by methylseleninic acid in human esophageal squamous cell carcinoma cells: Modification of histone H3 acetylation through HAT/HDAC interplay. Mol Carcinog. 2015; 54:1051–9. 10.1002/mc.22174. 24789055

[9] Wei Z , Gao F , Kim S , Yang H , Lyu J , An W , Wang K , Lu W . Klf4 organizes long-range chromosomal interactions with the oct4 locus in reprogramming and pluripotency. Cell Stem Cell. 2013; 13:36–47. 10.1016/j.stem.2013.05.010. 23747203

[10] Wang Y , Lu T , Sun G , Zheng Y , Yang S , Zhang H , Hao S , Liu Y , Ma S , Zhang H , Ru Y , Gao S , Yen K , et al. Targeting of apoptosis gene loci by reprogramming factors leads to selective eradication of leukemia cells. Nat Commun. 2019; 10:5594. 10.1038/s41467-019-13411-y. 31811153 PMC6898631

[11] Hu S , Wan J , Su Y , Song Q , Zeng Y , Nguyen HN , Shin J , Cox E , Rho HS , Woodard C , Xia S , Liu S , Lyu H , et al. DNA methylation presents distinct binding sites for human transcription factors. Elife. 2013; 2:e00726. 10.7554/eLife.00726. 24015356 PMC3762332

[12] Liu Y , Olanrewaju YO , Zheng Y , Hashimoto H , Blumenthal RM , Zhang X , Cheng X . Structural basis for Klf4 recognition of methylated DNA. Nucleic Acids Res. 2014; 42:4859–67. 10.1093/nar/gku134. 24520114 PMC4005678

[13] Feinberg MW , Wara AK , Cao Z , Lebedeva MA , Rosenbauer F , Iwasaki H , Hirai H , Katz JP , Haspel RL , Gray S , Akashi K , Segre J , Kaestner KH , et al. The Kruppel-like factor KLF4 is a critical regulator of monocyte differentiation. EMBO J. 2007; 26:4138–48. 10.1038/sj.emboj.7601824. 17762869 PMC2230668

[14] Park CS , Lee PH , Yamada T , Burns A , Shen Y , Puppi M , Lacorazza HD . Kruppel-like factor 4 (KLF4) promotes the survival of natural killer cells and maintains the number of conventional dendritic cells in the spleen. J Leukoc Biol. 2012; 91:739–50. 10.1189/jlb.0811413. 22345706 PMC3336774

[15] Mamonkin M , Shen Y , Lee PH , Puppi M , Park CS , Lacorazza HD . Differential roles of KLF4 in the development and differentiation of CD8+ T cells. Immunol Lett. 2013; 156:94–101. 10.1016/j.imlet.2013.09.008. 24075846 PMC3851948

[16] Yamada T , Park CS , Mamonkin M , Lacorazza HD . Transcription factor ELF4 controls the proliferation and homing of CD8+ T cells via the Kruppel-like factors KLF4 and KLF2. Nat Immunol. 2009; 10:618–26. 10.1038/ni.1730. 19412182 PMC2774797

[17] Park CS , Lewis A , Chen T , Lacorazza D . Concise Review: Regulation of Self-Renewal in Normal and Malignant Hematopoietic Stem Cells by Kruppel-Like Factor 4. Stem Cells Transl Med. 2019; 8:568–74. 10.1002/sctm.18-0249. 30790473 PMC6525558

[18] Li W , Jiang Z , Li T , Wei X , Zheng Y , Wu D , Yang L , Chen S , Xu B , Zhong M , Jiang J , Hu Y , Su H , et al. Genome-wide analyses identify KLF4 as an important negative regulator in T-cell acute lymphoblastic leukemia through directly inhibiting T-cell associated genes. Mol Cancer. 2015; 14:26. 10.1186/s12943-014-0285-x. 25644173 PMC4350611

[19] Shen Y , Park CS , Suppipat K , Mistretta TA , Puppi M , Horton TM , Rabin K , Gray NS , Meijerink JPP , Lacorazza HD . Inactivation of KLF4 promotes T-cell acute lymphoblastic leukemia and activates the MAP2K7 pathway. Leukemia. 2017; 31:1314–24. 10.1038/leu.2016.339. 27872496 PMC12831583

[20] Guan H , Xie L , Leithauser F , Flossbach L , Moller P , Wirth T , Ushmorov A . KLF4 is a tumor suppressor in B-cell non-Hodgkin lymphoma and in classic Hodgkin lymphoma. Blood. 2010; 116:1469–78. 10.1182/blood-2009-12-256446. 20519630

[21] Schoenhals M , Kassambara A , Veyrune JL , Moreaux J , Goldschmidt H , Hose D , Klein B . Kruppel-like factor 4 blocks tumor cell proliferation and promotes drug resistance in multiple myeloma. Haematologica. 2013; 98:1442–9. 10.3324/haematol.2012.066944. 23585530 PMC3762102

[22] Guo X , Tang Y . KLF4 translation level is associated with differentiation stage of different pediatric leukemias in both cell lines and primary samples. Clin Exp Med. 2013; 13:99–107. 10.1007/s10238-012-0187-4. 22592916

[23] Faber K , Bullinger L , Ragu C , Garding A , Mertens D , Miller C , Martin D , Walcher D , Dohner K , Dohner H , Claus R , Plass C , Sykes SM , et al. CDX2-driven leukemogenesis involves KLF4 repression and deregulated PPARgamma signaling. J Clin Invest. 2013; 123:299–314. 10.1172/JCI64745. 23202735 PMC3533294

[24] Somerville TD , Wiseman DH , Spencer GJ , Huang X , Lynch JT , Leong HS , Williams EL , Cheesman E , Somervaille TC . Frequent Derepression of the Mesenchymal Transcription Factor Gene FOXC1 in Acute Myeloid Leukemia. Cancer Cell. 2015; 28:329–42. 10.1016/j.ccell.2015.07.017. 26373280

[25] Valencia-Hipomicronlito A , Hernandez-Atenogenes M , Vega GG , Maldonado-Valenzuela A , Ramon G , Mayani H , Pena Alonso Y , Martinez-Maza O , Mendez-Tenorio A , Huerta-Yepez S , Bonavida B , Vega MI . Expression of KLF4 is a predictive marker for survival in pediatric Burkitt lymphoma. Leuk Lymphoma. 2014; 55:1806–14. 10.3109/10428194.2013.848437. 24067139

[26] Park CS , Lewis AH , Chen TJ , Bridges CS , Shen Y , Suppipat K , Puppi M , Tomolonis JA , Pang PD , Mistretta TA , Ma L , Green MR , Rau R , et al. A KLF4-DYRK2-mediated pathway regulating self-renewal in CML stem cells. Blood. 2019; 134:1960–72. 10.1182/blood.2018875922. 31515251 PMC6887114

[27] Tate JG , Bamford S , Jubb HC , Sondka Z , Beare DM , Bindal N , Boutselakis H , Cole CG , Creatore C , Dawson E , Fish P , Harsha B , Hathaway C , et al. COSMIC: the Catalogue Of Somatic Mutations In Cancer. Nucleic Acids Res. 2019; 47:D941–D7. 10.1093/nar/gky1015. 30371878 PMC6323903

[28] Morris VA , Cummings CL , Korb B , Boaglio S , Oehler VG . Deregulated KLF4 Expression in Myeloid Leukemias Alters Cell Proliferation and Differentiation through MicroRNA and Gene Targets. Mol Cell Biol. 2016; 36:559–73. 10.1128/MCB.00712-15. 26644403 PMC4751692

[29] Seipel K , Marques MT , Bozzini MA , Meinken C , Mueller BU , Pabst T . Inactivation of the p53-KLF4-CEBPA Axis in Acute Myeloid Leukemia. Clin Cancer Res. 2016; 22:746–56. 10.1158/1078-0432.CCR-15-1054. 26408402

[30] Ren M , Qin H , Wu Q , Savage NM , George TI , Cowell JK . Development of ZMYM2-FGFR1 driven AML in human CD34+ cells in immunocompromised mice. Int J Cancer. 2016; 139:836–40. 10.1002/ijc.30100. 27005999 PMC5754922

[31] Alder JK , Georgantas RW 3rd , Hildreth RL , Kaplan IM , Morisot S , Yu X , McDevitt M , Civin CI . Kruppel-like factor 4 is essential for inflammatory monocyte differentiation *in vivo* . J Immunol. 2008; 180:5645–52. 10.4049/jimmunol.180.8.5645. 18390749 PMC3074963

[32] Feinberg MW , Cao Z , Wara AK , Lebedeva MA , Senbanerjee S , Jain MK . Kruppel-like factor 4 is a mediator of proinflammatory signaling in macrophages. J Biol Chem. 2005; 280:38247–58. 10.1074/jbc.M509378200. 16169848

[33] Karpurapu M , Ranjan R , Deng J , Chung S , Lee YG , Xiao L , Nirujogi TS , Jacobson JR , Park GY , Christman JW . Kruppel like factor 4 promoter undergoes active demethylation during monocyte/macrophage differentiation. PLoS One. 2014; 9:e93362. 10.1371/journal.pone.0093362. 24695324 PMC3973678

[34] Kurotaki D , Osato N , Nishiyama A , Yamamoto M , Ban T , Sato H , Nakabayashi J , Umehara M , Miyake N , Matsumoto N , Nakazawa M , Ozato K , Tamura T . Essential role of the IRF8-KLF4 transcription factor cascade in murine monocyte differentiation. Blood. 2013; 121:1839–49. 10.1182/blood-2012-06-437863. 23319570 PMC3591803

[35] McCormack E , Micklem DR , Pindard LE , Silden E , Gallant P , Belenkov A , Lorens JB , Gjertsen BT . *In vivo* optical imaging of acute myeloid leukemia by green fluorescent protein: time-domain autofluorescence decoupling, fluorophore quantification, and localization . Mol Imaging. 2007; 6:193–204. 10.2310/7290.2007.00016. 17532885

[36] McCormack E , Mujic M , Osdal T , Bruserud O , Gjertsen BT . Multiplexed mAbs: a new strategy in preclinical time-domain imaging of acute myeloid leukemia. Blood. 2013; 121:e34–42. 10.1182/blood-2012-05-429555. 23243270

[37] Cercek A , Wheler J , Murray PE , Zhou S , Saltz L . Phase 1 study of APTO-253 HCl, an inducer of KLF4, in patients with advanced or metastatic solid tumors. Invest New Drugs. 2015; 33:1086–92. 10.1007/s10637-015-0273-z. 26268924

[38] Local A , Zhang H , Benbatoul KD , Folger P , Sheng X , Tsai CY , Howell SB , Rice WG . APTO-253 Stabilizes G-quadruplex DNA, Inhibits MYC Expression, and Induces DNA Damage in Acute Myeloid Leukemia Cells. Mol Cancer Ther. 2018; 17:1177–86. 10.1158/1535-7163.MCT-17-1209. 29626127

[39] Ley TJ , Miller C , Ding L , Raphael BJ , Mungall AJ , Robertson A , Hoadley K , Triche TJ Jr , Laird PW , Baty JD , Fulton LL , Fulton R , Heath SE , et al, and Cancer Genome Atlas Research Network. Genomic and epigenomic landscapes of adult de novo acute myeloid leukemia. N Engl J Med. 2013; 368:2059–74. 10.1056/NEJMoa1301689. 23634996 PMC3767041

[40] Papaemmanuil E , Gerstung M , Bullinger L , Gaidzik VI , Paschka P , Roberts ND , Potter NE , Heuser M , Thol F , Bolli N , Gundem G , Van Loo P , Martincorena I , et al. Genomic Classification and Prognosis in Acute Myeloid Leukemia. N Engl J Med. 2016; 374:2209–21. 10.1056/NEJMoa1516192. 27276561 PMC4979995

[41] Gerstung M , Jolly C , Leshchiner I , Dentro SC , Gonzalez S , Rosebrock D , Mitchell TJ , Rubanova Y , Anur P , Yu K , Tarabichi M , Deshwar A , Wintersinger J , et al. The evolutionary history of 2,658 cancers. Nature. 2020; 578:122–8. 10.1038/s41586-019-1907-7. 32025013 PMC7054212

[42] Chen L , Liu S , Tao Y . Regulating tumor suppressor genes: post-translational modifications. Signal Transduct Target Ther. 2020; 5:90. 10.1038/s41392-020-0196-9. 32532965 PMC7293209

[43] Esteller M . CpG island hypermethylation and tumor suppressor genes: a booming present, a brighter future. Oncogene. 2002; 21:5427–40. 10.1038/sj.onc.1205600. 12154405

[44] Borgonovo T , Ribeiro EM , Cornélio DA , Schmid-Braz DT , Jamur VR , Wuicik L , Acosta Veiga LB , Ehmke NAM , Pasquini R , Cavalli IJ . Cytogenetic study of Brazilian patients with myelodysplastic syndrome (MDS). Genetics and Molecular Biology. 2005; 28 10.1590/s1415-47572005000500002.

[45] Chen ZY , Wang X , Zhou Y , Offner G , Tseng CC . Destabilization of Kruppel-like factor 4 protein in response to serum stimulation involves the ubiquitin-proteasome pathway. Cancer Res. 2005; 65:10394–400. 10.1158/0008-5472.CAN-05-2059. 16288030

[46] Gamper AM , Qiao X , Kim J , Zhang L , DeSimone MC , Rathmell WK , Wan Y . Regulation of KLF4 turnover reveals an unexpected tissue-specific role of pVHL in tumorigenesis. Mol Cell. 2012; 45:233–43. 10.1016/j.molcel.2011.11.031. 22284679 PMC3982234

[47] Morita K , Masamoto Y , Kataoka K , Koya J , Kagoya Y , Yashiroda H , Sato T , Murata S , Kurokawa M . BAALC potentiates oncogenic ERK pathway through interactions with MEKK1 and KLF4. Leukemia. 2015; 29:2248–56. 10.1038/leu.2015.137. 26050649

[48] Haferlach T , Bacher U , Kern W , Schnittger S , Haferlach C . Diagnostic pathways in acute leukemias: a proposal for a multimodal approach. Ann Hematol. 2007; 86:311–27. 10.1007/s00277-007-0253-2. 17375301

[49] Bagger FO , Kinalis S , Rapin N . BloodSpot: a database of healthy and malignant haematopoiesis updated with purified and single cell mRNA sequencing profiles. Nucleic Acids Res. 2019; 47:D881–D5. 10.1093/nar/gky1076. 30395307 PMC6323996

[50] Bagger FO , Sasivarevic D , Sohi SH , Laursen LG , Pundhir S , Sonderby CK , Winther O , Rapin N , Porse BT . BloodSpot: a database of gene expression profiles and transcriptional programs for healthy and malignant haematopoiesis. Nucleic Acids Res. 2016; 44:D917–24. 10.1093/nar/gkv1101. 26507857 PMC4702803

[51] Zhao Z , Goldin L , Liu S , Wu L , Zhou W , Lou H , Yu Q , Tsang SX , Jiang M , Li F , McMaster M , Li Y , Lin X , et al. Evolution of multiple cell clones over a 29-year period of a CLL patient. Nat Commun. 2016; 7:13765. 10.1038/ncomms13765. 27982015 PMC5171825

[52] Lichtman MA . A historical perspective on the development of the cytarabine (7 days) and daunorubicin (3 days) treatment regimen for acute myelogenous leukemia: 2013 the 40th anniversary of 7+3. Blood Cells Mol Dis. 2013; 50:119–30. 10.1016/j.bcmd.2012.10.005. 23154039

[53] Oran B , Weisdorf DJ . Survival for older patients with acute myeloid leukemia: a population-based study. Haematologica. 2012; 97:1916–24. 10.3324/haematol.2012.066100. 22773600 PMC3590098

