# Supplementary material for: Krüppel-like factor 4 promotes survival and expansion in acute myeloid leukemia cells
Source: Oncotarget. 2021 Feb 16;12(4):255–67. doi: 10.18632/oncotarget.27878 (PMC7899553; doi:10.18632/oncotarget.27878)
Supplement: Supplementary file 1 [file oncotarget-12-255-s001.pdf]

Krüppel-like factor 4 promotes survival and expansion in acute myeloid leukemia cells

SUPPLEMENTARY MATERIALS

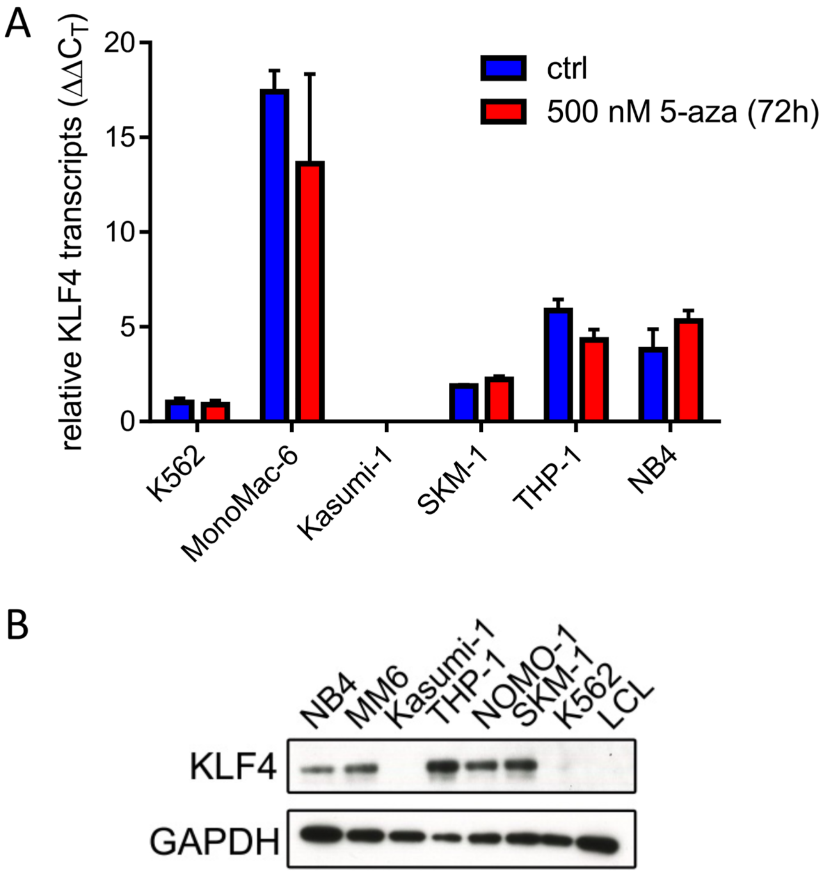

Supplementary Figure 1: mRNA and protein expression of KLF4 in AML cell lines. (**A**) Relative KLF4 expression by qPCR of AML and CML cells cultured in the presence and absence of 500 nM 5-Aza for 72 hours to induce gene demethylation. *Data is expressed as mean ± s.d. of* $\Delta\Delta C_T$ ($n = 3$). (**B**) Protein expression of KLF4 in a Panel of AML, CML, and LCL cells detected by immunoblot.

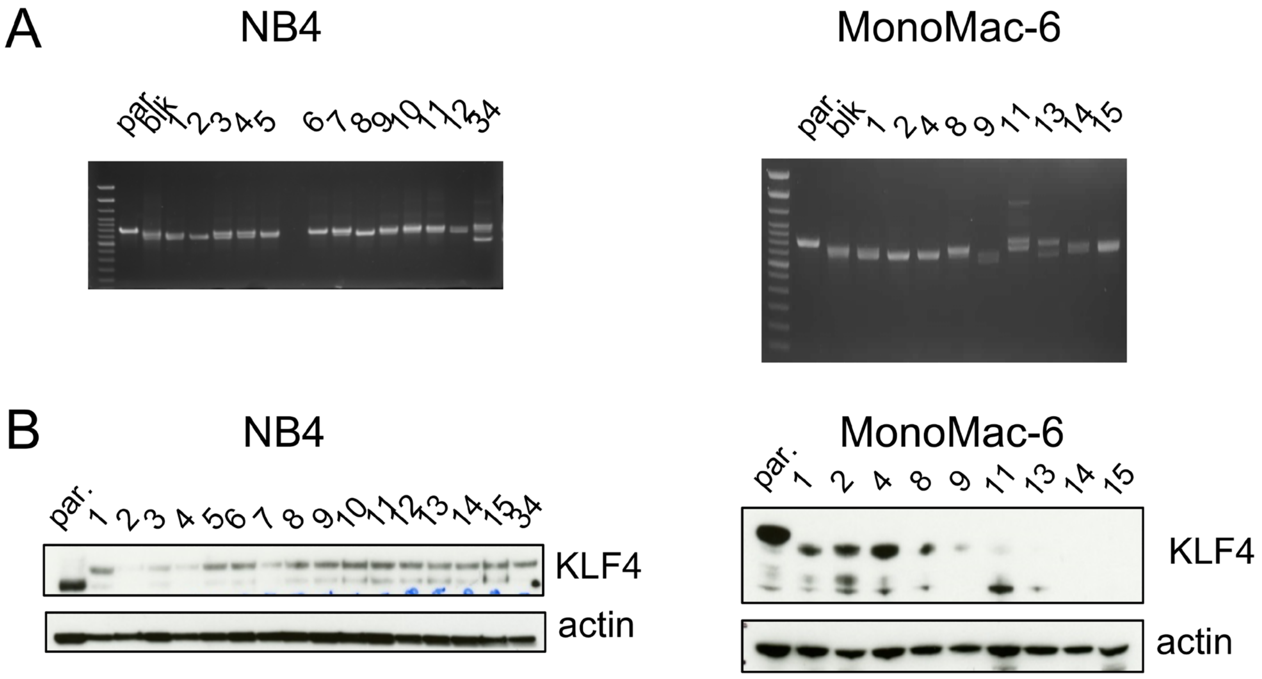

Supplementary Figure 2: Validation of Cas9-based deletion of KLF4 gene in NB4 and MM6 cell clones. (**A**) PCR of intact (~800 bp) *KLF4* genomic region in parental, bulk and single-cell clones of NB4 and MonoMac-6 cells. (**B**) Immunoblot analysis of KLF4 protein levels in parental and single-cell clones of NB4 and MM6 cells.

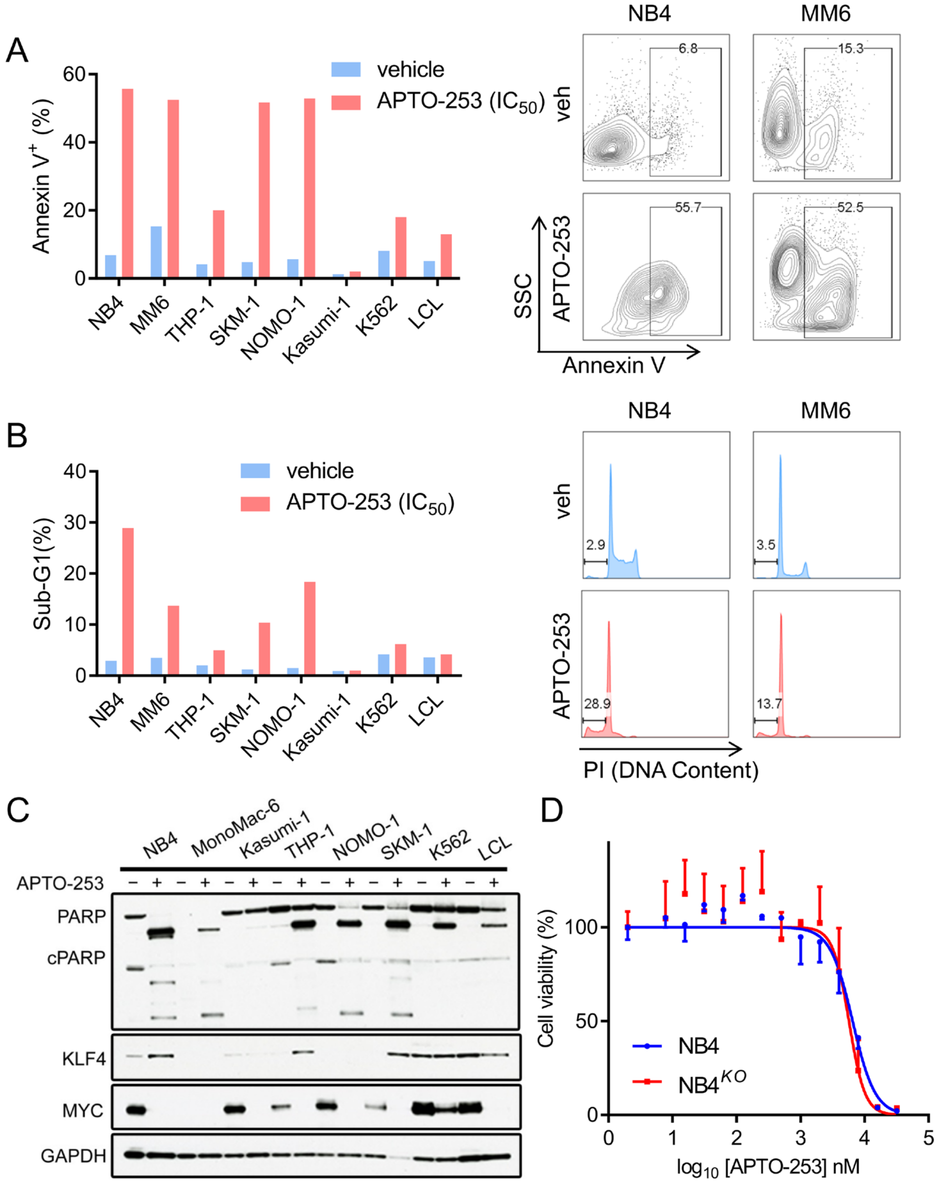

Supplementary Figure 3: APTO-253 induces apoptosis in NB4 cells independently of KLF4. (**A**) Flow cytometric analysis of Annexin V in AML cells after treatment with IC_{50} concentration of APTO-253. Plots of NB4 and MM6 shown. (**B**) Flow cytometric analysis of sub-G_1 DNA content in AML cells after treatment with IC_{50} concentration of APTO-253. Plots of NB4 and MM6 shown. (**C**) Immunoblot showing full length and cleaved PARP in AML cells after treatment with IC_{50} concentration of APTO-253 for 48 hours. Expression of KLF4, c-Myc, and GAPDH was also evaluated. (**D**) Cytotoxicity of of NB4 and $NB4^{KO}$ cells to increasing doses of APTO-253. Data represent mean ± s.d. (*n* = 3). Data are representative of 2 independent experiments.

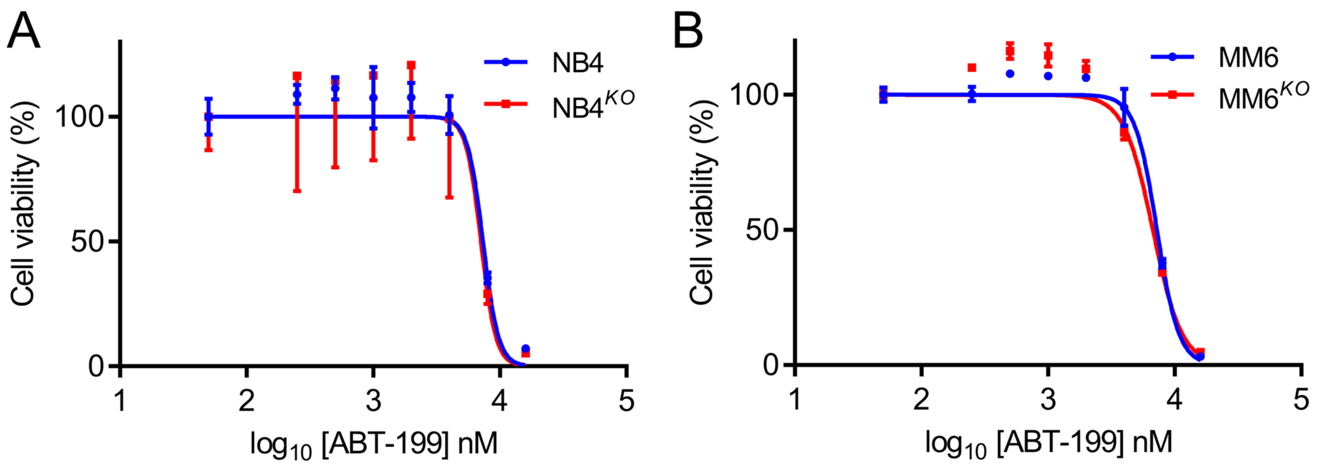

Supplementary Figure 4: KLF4 deletion does not alter sensitivity to ABT-199 (venetoclax) in AML cells. Cytotoxicity of NB4 and NB4^{KO} (**A**) and MM6 and MM6^{KO} (**B**) to increasing log doses of ABT-199. Determined using Cell-Titer Glo cytotoxicity assay after 48h treatment. Data represent mean ± s.d. (*n* = 3).
